# Clinical Efficacy and Pharmacokinetics of Antivenom Viperfav^®^ in *Vipera ammodytes ammodytes* Envenomation

**DOI:** 10.3390/pharmaceutics17111431

**Published:** 2025-11-05

**Authors:** Tihana Kurtović, Mojca Dobaja Borak, Damjan Grenc, Adrijana Leonardi, Igor Križaj, Boris Lukšić, Beata Halassy, Miran Brvar

**Affiliations:** 1 Centre for Research and Knowledge Transfer in Biotechnology, University of Zagreb, Rockefellerova 10, HR-10000 Zagreb, Croatia; tihana.kurtovic@unizg.hr (T.K.); bhalassy@unizg.hr (B.H.); 2 Centre for Clinical Toxicology and Pharmacology, University Medical Centre Ljubljana, Zaloška cesta 7, SI-1000 Ljubljana, Slovenia; mojca.dobaja@kclj.si (M.D.B.); damjan.grenc@kclj.si (D.G.); 3 Faculty of Medicine, University of Ljubljana, Vrazov trg 2, SI-1000 Ljubljana, Slovenia; 4 Department of Molecular and Biomedical Sciences, Jožef Stefan Institute, Jamova 39, SI-1000 Ljubljana, Slovenia; adrijana.leonardi@ijs.si (A.L.); igor.krizaj@ijs.si (I.K.); 5 Clinical Department of Infectious Diseases, University Hospital of Split, Šoltanska 1, HR-21000 Split, Croatia; luksic.boris01@gmail.com; 6 School of Medicine, University of Split, Šoltanska 2, HR-21000 Split, Croatia; 7 Centre for Clinical Physiology, Faculty of Medicine, University of Ljubljana, Zaloška cesta 4, SI-1000 Ljubljana, Slovenia

**Keywords:** snakebite, antivenom, *V. ammodytes*, Viperfav, F(ab’)_2_ fragments, pharmacokinetics

## Abstract

**Background:** In Europe, *Vipera ammodytes ammodytes* (*Vaa*, nose-horned viper) is considered the most venomous of the European vipers. The antivenom Viperfav^®^, composed of polyvalent equine F(ab′)_2_ fragments, is effective against *Vipera aspis*, *Vipera berus* and *Vaa*. **Objectives**: This study aimed to evaluate the clinical efficacy and pharmacokinetics of Viperfav in *Vaa* envenomations. **Methods:** Patients presenting with *Vaa* snakebite and treated with intravenous Viperfav were included. Clinical manifestations and laboratory findings were assessed on admission to the Emergency Department, prior to antivenom therapy, and monitored throughout hospitalization. Blood samples were collected on arrival and at defined intervals after Viperfav administration. Venom and antivenom concentrations in serum were determined by ELISA and subjected to pharmacokinetic analysis. **Results:** Twenty-one patients bitten by *Vaa* and classified with a severity score of 2b on the modified Audebert clinical severity scale received a single intravenous dose of Viperfav within 4 h of the bite. Viperfav attenuated the progression of local symptoms and prevented the development of new systemic manifestations. The serum concentrations of F(ab′)_2_ fragments reached 196 µg/mL, far exceeding the venom concentration at admission (35 ng/mL). The prolonged elimination half-life of Viperfav (49 h) corresponded with the absence of recurrent symptoms after a single dose. Bradycardia or hypotension occurred in 10% of patients; no cases of anaphylaxis or serum sickness were observed. **Conclusions:** A single intravenous dose of Viperfav demonstrated clinical efficacy and a favourable pharmacokinetic profile in *Vaa* envenomed patients when administered within hours of the bite.

## 1. Introduction

In Europe, *Vipera ammodytes ammodytes* (*Vaa*, the nose-horned viper) is considered the most venomous of all European vipers, owing to its considerable size (up to 95 cm in length), elongated fangs (reaching up to 13 mm), and potent venom containing haematotoxic and neurotoxic components [1]. This species inhabits Southern Europe, with its range extending across the Balkan Peninsula, including both the coastal and central regions of Slovenia (e.g., Ljubljana). It is easily distinguished from *Vipera berus* (*V. berus*), which is found in the alpine regions of Slovenia, by the presence of its distinctive nasal horn.

*Vaa* venomous bites have recently been treated with Viperfav^®^ (MicroPharm Ltd., UK). The antivenom Viperfav contains polyvalent equine F(ab′)_2_ fragments effective against *Vipera aspis* (*V. aspis*), *V. berus*, and *Vaa* venom components. Pharmacokinetics of Viperfav antivenom in patients envenomed by *Vaa* has not yet been described, although protective efficacy has been proven preclinically [2,3].

The aim of this study was to evaluate clinical efficacy and pharmacokinetics of Viperfav in *Vaa* envenomations.

## 2. Materials and Methods

This prospective study included consecutive patients who sustained envenomation by *Vaa* and were treated with Viperfav at the University Medical Centre Ljubljana, Slovenia, between 2020 and 2024.

The University Medical Centre Ljubljana is a tertiary referral hospital located in the Slovenian capital, serving a local population of approximately 600,000 and a national population of about two million. The study protocol was reviewed and approved by the Slovenian National Medical Ethics Committee (Approval No. 87/07/15).

### 2.1. Patients

Patients envenomed by *Vaa* and treated with the antivenom Viperfav were included in the study. Cases were eligible only if *Vaa* envenomation was confirmed either by the detection of *Vaa* venom-specific ammodytoxins (Atxs) in the patient’s serum or by photographic identification of the snake in situ.

Data were collected prospectively and included demographic information (age, sex), circumstances of the bite (anatomical location and time of snakebite), local manifestations (pain, oedema, ecchymosis, lymphadenitis), systemic manifestations (nausea, vomiting, diarrhea, dizziness, syncope, level of consciousness, cranial nerve palsies, pulse rate, systolic blood pressure), laboratory parameters (myoglobin, creatine kinase, creatinine, troponin I, liver function tests, international normalized ratio (prothrombin time), activated partial thromboplastin time, D-dimer, fibrinogen, platelet count, C-reactive protein, procalcitonin, leukocyte count, and lactate), treatment details (type of medication, timing, dosage, adverse effects), and clinical outcomes (length of hospital stay, mortality).

Clinical signs and laboratory results were recorded at presentation to the ED before antivenom administration and reassessed throughout hospitalization. Final or peak clinical symptoms and laboratory values were documented as the most severe, lowest, or highest values observed during hospitalization, regardless of the timing of antivenom administration. All patients were instructed to return for evaluation in case of delayed symptoms such as arthralgia.

Tachycardia was defined as a heart rate exceeding 100 beats per minute (bpm), hypotension as a systolic blood pressure below 90 mmHg, and shock as the presence of hypotension accompanied by elevated serum lactate concentration. Cranial nerve palsies included ptosis, ophthalmoplegia, or dysphagia. Acute kidney injury was diagnosed when serum creatinine levels were at least twice the upper limit of normal. Rhabdomyolysis was defined by serum myoglobin and creatine kinase levels at least twice the upper normal limit. Acute respiratory failure was diagnosed in patients with tachypnoea (>25 breaths per minute) and partial pressure of oxygen (PaO_2_) below 8 kPa. Acute myocardial injury was defined as a positive troponin I Ultra (>0.10 µg/L) or troponin I High sensitivity (>60 ng/L). Leukocytosis and thrombocytopenia were defined as leukocyte counts above 11 × 10^9^/L and platelet counts below 150 × 10^9^/L, respectively. Disseminated intravascular coagulation (DIC) was diagnosed in patients presenting with thrombocytopenia, elevated D-dimer, prolonged prothrombin time (INR) and activated partial thromboplastin time, and reduced fibrinogen concentration.

Patients were classified according to the modified Audebert clinical severity score, which includes neurotoxic manifestations and atypical systemic symptoms not considered in the original version [4], making it more suitable for assessing *Vaa* envenomation. Each patient’s severity grade was determined by the treating team, composed of two or three physicians, who reached a consensus regarding both grading and the decision to administer antivenom.

### 2.2. Antivenom Therapy

Patients received an initial dose of 4 mL of Viperfav, containing F(ab′)_2_ fragments at a concentration of 99–116 mg/mL, diluted in 100 mL of 0.9% NaCl and administered intravenously over 60 min. Indications for antivenom therapy included progression of oedema beyond a major joint (involving the arm or thigh) or the presence of systemic manifestations of *Vaa* envenomation. Antivenom was administered in the ED and/or the Centre for Clinical Toxicology and Pharmacology.

Supplemental doses were administered using the same protocol when clinically indicated, such as further progression of local swelling or deterioration in laboratory parameters, particularly thrombocytopenia. Supportive therapy was provided according to the patient’s clinical condition and laboratory findings.

### 2.3. Blood Samples

Blood samples were collected in serum tubes upon admission to the emergency department, at the initiation of the 60 min Viperfav infusion, and subsequently at 0, 30 min, 1, 2, 4, 6, 12, and 24 h after antivenom administration. Sampling was continued at 12 h intervals until hospital discharge. All samples were immediately centrifuged, aliquoted, and stored at –50 °C until analysis for venom and antivenom quantification.

### 2.4. Reagents and Chemicals

Horseradish peroxidase-conjugated rabbit anti-guinea pig IgG, HRP-conjugated rabbit anti-equine IgG, bovine serum albumin (BSA), Tween 20, and *o*-phenylenediamine dihydrochloride (OPD) were obtained from Sigma-Aldrich (St. Louis, MO, USA). HRP-conjugated goat anti-equine F(ab′)_2_ IgG was from Antibodies Online (Aachen, Germany). Buffer reagents were supplied by AppliChem (Aachen, Germany). *Vaa* venom was provided by the Institute of Immunology Inc. (Zagreb, Croatia). Recombinant Atx was produced as described by Lian et al. [5]. Viperfav was obtained from MicroPharm Ltd. (Newcasle Emlyn, UK).

### 2.5. Quantification of Vaa Venom in Sera Samples

A microtiter plate was coated with in-house rabbit anti-*Vaa* venom IgG (100 μL/well, 5 μg/mL) in 0.05 M carbonate buffer, pH 9.6, and incubated overnight at room temperature (RT). The plate was then blocked with 2% (*w*/*v*) BSA in PBS containing 0.05% (*v*/*v*) Tween 20 (PBST buffer) (250 μL/well) for 4 h at 37 °C. Patient sera were appropriately diluted in 0.5% (*m*/*v*) BSA in PBST buffer, added in duplicate (100 μL/well), and incubated overnight at RT. For the standard curve, whole venom was serially diluted 1.5-fold (eight points), starting at 15 ng/mL, in 0.5% (*w*/*v*) BSA in PBST buffer containing the corresponding percentage (*v*/*v*) of pooled sera from non-envenomed individuals (normal serum), and added in duplicate (100 μL/well). Normal serum, diluted identically to the patient samples, was used as a negative control. After overnight incubation on RT, the plate was incubated with in-house horse anti-*Vaa* venom IgG (100 μL/well, 5.7 μg/mL), followed by rabbit HRP-conjugated anti-equine IgG (100 μL/well, 1:4000 dilution). Between each incubation step, the plate was washed extensively. OPD substrate solution (5.5 mM in 0.15 M citrate-phosphate buffer, pH 5.0, with 0.5 μL/mL of 30% H_2_O_2_) was added and incubated for 30 min at RT in the dark. The reaction was stopped with 1 M H_2_SO_4_ (50 μL/well), and absorbance was measured at 492 nm. Venom concentrations were calculated from the standard curve and adjusted by the corresponding dilution factor.

The cut-off value was the mean absorbance of the normal serum, which was analyzed in the same dilution used for venom-containing samples, plus three standard deviations. Samples with absorbance values below this threshold were assigned a concentration of zero ng/mL.

### 2.6. Quantification of Atxs in Sera Samples

ELISA for the detection of Atxs was performed similarly to the whole venom assay, with a few modifications. A plate was coated with in-house rabbit anti-Atx IgG (100 μL/well, 1 μg/mL) and incubated as described above. After washing and blocking, patient sera (diluted appropriately depending on the individual) and pure Atx standard (eight serial 1.5-fold dilutions starting from 1.5 ng/mL in the respective matrix) were added in duplicate. Following washing, the plate was first incubated with in-house guinea pig anti-Atx IgG (100 μL/well, 1.5 μg/mL), and then with rabbit HRP-conjugated anti-guinea pig IgG (100 μL/well, 1:10,000 dilution). The remaining steps were performed as described in the previous section.

### 2.7. Quantification of Antivenom in Sera Samples

For determination of antivenom, a plate was coated with *Vaa* venom (100 μL/well, 1 μg/mL) in 0.05 M carbonate buffer, pH 9.6, and incubated overnight at RT. After washing, wells were blocked with 0.5% (*w*/*v*) BSA in PBST buffer (200 μL/well) for 2 h at 37 °C. Patient sera were added in duplicate across a suitable range of 2-fold dilutions (100 μL/well), together with Viperfav (starting at 250 ng/mL), which served as the standard for quantification of F(ab′)_2_ fragments, and incubated overnight at RT. The plate was then washed and incubated with goat HRP-conjugated anti-equine F(ab′)_2_ IgG (100 μL/well, 1:5000 dilution). The final steps were performed as described in Section 2.5.

### 2.8. Pharmacokinetic Analysis

Pharmacokinetic analysis of the measured concentrations was carried out using the PKSolver add-in for Microsoft Excel (version 2.0, China Pharmaceutical University, Nanjing, China) [6]. Non-compartmental analysis following intravenous constant infusion input was applied to determine the pharmacokinetic parameters.

### 2.9. Statistical Analysis

Data are presented as median and interquartile range (IQR) for continuous variables and as the frequency (percentage) for categorical variables. Odds ratios (OR) with Haldane-Anscombe correction and the corresponding confidence interval are given for categorical variables. The McNemar’s test was used for categorical variables, and the Wilcoxon signed-rank test for paired continuous data to detect differences between on admission and during hospitalization. A *p*-value of 0.05 was considered significant. Analyses were carried out with the Statistical Package for Social Science 23 for Windows (SPSS).

## 3. Results

During the study period, 21 patients bitten by *Vaa* and classified with a severity score of 2b on the modified Audebert clinical severity scale (maximum score: 3) were admitted to the University Medical Centre Ljubljana. All received Viperfav and met the inclusion criteria. Their general characteristics are summarized in Table 1. No *Vaa*-bitten patient treated with Viperfav was excluded.

Table 2 presents the serum venom concentrations, clinical signs and symptoms observed on admission to the emergency department, as well as the most severe manifestations and peak laboratory values recorded during envenomation and antivenom therapy.

All patients received a single intravenous dose (4 mL) of Viperfav within 3.4 to 5.6 h after the bite (Table 3). No patient needed the second dose. Following Viperfav administration, 2 of the 21 patients experienced transient, mild, asymptomatic bradycardia (down to 40 beats per minute) or asymptomatic hypotension (as low as 90/40 mmHg) within half an hour after completion of the Viperfav infusion, requiring no additional therapy. No cases of anaphylaxis or serum sickness were reported (Table 3). Two patients received antihistamines and corticosteroids administered by emergency physicians prior to admission. Antiemetics and analgesics were given to 12 patients treated with Viperfav (Table 3).

An intravenous dose of Viperfav (4 mL), administered a median of 4 h after the bite and approximately 1.3 h after emergency department admission, resulted in a peak serum concentration of 196.4 µg/mL (135.6–268.2); see Table 4. Additional pharmacokinetic parameters of the *Vaa* venom-specific equine F(ab′)_2_ fragments (Viperfav) after intravenous administration in 21 envenomed patients are presented in Table 4. The reported maximum serum F(ab′)_2_ concentrations correspond to the first post-infusion blood sample; however, the actual *c*_max_ probably occurred at the end of the 1 h Viperfav infusion.

## 4. Discussion

This study demonstrates that intravenous administration of Viperfav is clinically effective in patients envenomed by *Vaa*, as it attenuates the progression of local symptoms and prevents new systemic manifestations, with the exception of local lymphadenitis. The pharmacokinetic profile supports this efficacy, with the high serum concentrations of F(ab′)_2_ fragments (196 µg/mL) far exceeding the venom concentration (35 ng/mL) determined on admission of the patients at the ED. Furthermore, the prolonged elimination half-life of Viperfav (49 h) explains the absence of recurrent symptoms after a single dose, in contrast to the short half-life of European viper venom (approximately 8 h) [7].

In this study, local swelling remained limited to the affected limb following Viperfav administration; however, the progression of swelling within the affected limb was not systematically documented, apart from proximal extension toward the trunk and the appearance of ecchymosis, which was unusual and not statistically significant, and did not require additional symptomatic treatment or repeated antivenom administration. In addition, no patient developed new systemic symptoms of envenomation after antivenom administration. These results highlight that early administration, within 4 h of the bite, has a favourable impact on clinical outcomes. They are consistent with the studies on European viper envenomations in France and Sweden showing that Viperfav effectively limits swelling progression and resolves systemic symptoms within 5 h of Viperfav infusion [8,9]. However, despite Viperfav treatment, patients in this study developed painful regional lymphadenitis, affecting lymph nodes in either the axillary or inguinal regions. The observed lymphadenitis likely reflects a local inflammatory response and tissue injury caused by ongoing local venom activity [10,11]. Moreover, intravenous antivenom administration may not fully prevent venom dissemination via local lymphatic pathways, as demonstrated in a sheep model of *Vaa* envenomation following intravenous administration of F(ab′)_2_ fragments [12].

Furthermore, in patients with laboratory abnormalities such as rhabdomyolysis, coagulopathy, or elevated inflammatory markers on admission, Viperfav did not completely prevent their progression, with the exception of thrombocytopenia. These changes were clinically insignificant and probably reflected a systemic inflammatory response syndrome, persistent coagulopathy and the release of muscular enzymes as a result of local tissue damage. Consequently, no patient required additional symptomatic interventions or readministration of antivenom dosing based on the laboratory results. Delayed improvement in laboratory parameters has also been reported in *V. aspis* and *V. berus* envenomations treated with Viperfav [13,14]; however, values usually normalized within 24 h following the bite and Viperfav administration [9]. In contrast, a French study on *V. aspis* and *V. berus* envenomations demonstrated that Viperfav corrected all systemic signs and laboratory abnormalities within 6–12 h [15]. A limitation of the present study is that symptoms and laboratory parameters were not continuously monitored; instead, only admission values and the most severe recorded results were assessed. Furthermore, the resolution time of local symptoms could not be determined, as patients were discharged before complete resolution of local signs such as oedema and pain. The relatively small sample size also limits the statistical power of the study, and outcomes might differ in a larger cohort; however, this reflects the low incidence of snakebites in Europe. The interpretation of certain non-significant findings such as oedema spreading to the trunk, ecchymosis, and changes in D-dimer, procalcitonin, and leukocyte counts is further constrained by high *p*-values and wide confidence intervals (Table 2), suggesting that the absence of significant differences may result from the limited sample size rather than a true lack of effect. In addition, a comparison with the clinical course of *Vaa* envenomation without antivenom therapy was not feasible, as all patients with grade 2b envenomation required antivenom treatment. Therefore, the results should be interpreted with caution, as they may not be fully generalizable to other settings or populations.

Overall, no patient in this study progressed to a higher severity grade after Viperfav administration. This observation is consistent with findings from mainland France, where progression occurred in only 0.5% of patients treated with Viperfav [4]. Based on these data, Boels et al. recommend the earliest possible administration of antivenom in grade 2a envenomations to minimize the risk of progression in *V. berus* and *V. aspis* bites [4,15]. In contrast, all patients in the present series of *Vaa* envenomations already had a severity grade 2b on admission, with a median delay of 2.5 h post-bite. This difference may be due to the larger size and higher venom yield of the *Vaa* compared to other European vipers.

Data on the pharmacokinetics of antivenoms in snakebite envenomation—particularly those containing F(ab′)_2_ fragments—remain limited [16,17,18,19]. Viperfav is no exception, despite its long-standing and widespread use for the treatment of *V. aspis* and *V. berus* bites [9,15,20,21]. To date, only one case report has described the use and pharmacokinetics of Viperfav in *Vaa* envenomation [22]. In contrast, pharmacokinetic studies have been published for ViperaTAb (Fab) and Zagreb antivenom (F(ab′)_2_) in *Vaa* envenomation, but not for *V. berus* or *V. aspis* envenomations [23]. Both antivenoms contain antibody fragments; Zagreb antivenom—like Viperfav—contains F(ab′)_2_ fragments but is administered intramuscularly. Intramuscular administration of F(ab′)_2_ fragments led to a gradual increase in serum antivenom concentrations, reaching peak levels approximately 120 h post-administration. This slow absorption necessitates early dosing; however, the prolonged elimination half-life of up to 317 h makes additional doses unnecessary [23].

The pharmacokinetics of Viperfav in *Vaa* envenomation in this study are summarized in Table 4. Considerable inter-individual variability was observed within the analyzed group, which is not unexpected given the impact of population-related factors such as gender, age, genetic variability, comorbidities, body weight, and environmental influences [24]. Despite this variability, the pharmacokinetic parameters determined in this study were consistent with those reported for other intravenously administered F(ab′)_2_-based antivenoms, which generally have a half-life of 40 to 100 h [15]. Of the antivenoms containing F(ab′)_2_ fragments with documented pharmacokinetic profiles, Viperfav most closely resembled the Russell’s viper antivenom, particularly with respect to elimination half-life (49.5 vs. 36 h) [19]. For the Government Pharmaceutical Organization of Thailand (GPO) and Indian snake antivenoms, the reported elimination half-life values were about three- and four-fold higher, respectively [16,17], while the elimination half-life of the Ipser Africa antivenom was more than twice as short [18]. The observed inconsistency may be attributed to differences in the quantification methods and pharmacokinetic models used, as well as to variations in the administration rate and sampling schedule.

All of these may have contributed to the discrepant clearance values (CL) reported for Viperfav (0.5 (mL/h)/kg) compared to GPO (1.7 (mL/h)/kg) or Indian snake antivenom (1.4 (mL/h)/kg) [16,17]. Additionally, Viperfav demonstrated a markedly higher *c*_max_ (196 µg/mL) than GPO (18.5 µg/mL) [17], likely due to the different dosing of the antivenom. Interestingly, despite the much lower dosage (400 vs. 2120 mg), Viperfav had an AUC value (5002.4 µg/mL∙h) which was comparable to that of Indian snake venom (9836.8 µg/mL∙h) [18], suggesting possible differences in bioavailability or systemic retention. These discrepancies may result from variations in F(ab′)_2_ fragment properties (e.g., affinity, specificity) [25,26] or from patient-related pathophysiological factors, such as increased capillary permeability [27,28]. Venom-induced capillary leak and increased vascular permeability have been documented in viperid envenomation, which may alter the distribution and clearance of antivenom in patients [27,28].

All the above-mentioned inter-study variations highlight the importance of conducting a standardized, comparative pharmacokinetic study in which all antivenoms of interest are investigated under uniform experimental conditions. Such an approach would allow for a more accurate assessment of their relative pharmacokinetic properties and therapeutic potential, minimizing methodological biases associated with dosing, sampling schedules, quantification procedures, and pharmacokinetic modelling.

Comparative data from other pharmacokinetic studies provide a valuable methodological and conceptual framework. It has been demonstrated that altered physiological conditions can significantly affect key pharmacokinetic parameters such as AUC, *c*_max_, and *t*_max_ [29]. Similarly, host-related factors, including co-medication, have been shown to markedly influence drug disposition and pharmacokinetic parameters such as AUC and *c*_max_ [30,31]. These findings are relevant to antivenom pharmacokinetics, as patient-specific factors, including comorbidities, concurrent therapies, and altered physiological states, may likewise affect Viperfav distribution and clearance.

No serious adverse events related to Viperfav were documented in this study. Asymptomatic, transient mild hypotension occurred in one patient, and mild asymptomatic bradycardia was observed in the patient with a high venom burden after Viperfav application. No cases of anaphylaxis or serum sickness were noted, corroborating previous safety data in *V. aspis* envenomation [4,9].

All patients survived, with a median hospital stay of 2.5 days. These results suggest that a single intravenous dose of Viperfav in combination with symptomatic treatment—such as antiemetics, analgesics, and intravenous hydration—is generally sufficient for effective management of *Vaa* envenomation. No benefit of multiple Viperfav doses has been demonstrated in clinical studies [15], and both the clinical outcomes and pharmacokinetic parameters reported here do not support repeated dosing.

## 5. Conclusions

A single intravenous dose of Viperfav demonstrated clinical efficacy and a favourable pharmacokinetic profile in *Vaa* envenomated patients classified as grade 2b on the modified Audebert clinical severity scale when administered within a few hours post-bite. One dose is generally sufficient; however, patients should remain under close clinical monitoring.

## Figures and Tables

**Table 1:** General characteristics of *Vaa* envenomed patients.

Age (median, IQR) (year)	32 (20–69)
Gender (male)	14
Weight (median, IQR) (kg)	81 (70–90)
Comorbidities	6/21
Bite location	
arm	20/21
leg	1/21
Time from bite to admission at the ED (h)	2.5 (2.0–3.5)

Legend: ED—Emergency Department; IQR—interquartile range.

**Table 2:** Clinical presentation prior to antivenom administration and the most severe symptoms and peak laboratory values during *Vaa* envenomation and antivenom therapy.

	*Vaa* Envenomed Patients’ Characteristics on Admission Before Antivenom Application (*n* = 21)	The Most Severe Symptoms and Peak Laboratory Results During *Vaa* Envenomation and Antivenom Therapy (*n* = 21)	OR (95% CI)	*p*
Time from bite to venom concentration measurement (h)	3.3 (2.1–4.6)	3.5 (2.4–7.0)		0.07
Venom concentration (ng/mL)	35.7 (12.4–56.4)	37.5 (13.9–56.7)		0.07
Atxs concentration (ng/mL)	1.2 (0.8–6.1)	2.5 (0.8–8.3)		0.32
Local pain	21/21	21/21	1 (0.01–52.7)	1.00
Local oedema	21/21	21/21	1 (0.01–53.1)	1.00
Local lymphadenitis	0/21	12/21	56.6 (3.0–1057.6)	0.01
Oedema spread to trunk	0/21	5/21	14.3 (0.7–278.1)	0.06
Ecchymosis	6/21	11/21	2.8 (0.8–9.9)	0.06
Nausea	11/21	11/21	1 (0.3–3.6)	1.00
Vomiting	7/21	7/21	1 (0.3–3.6)	1.00
Diarrhea	6/21	6/21	1 (0.3–3.8)	1.00
Abdominal pain	4/21	4/21	1 (0.2–4.7)	1.00
Tachycardia	4/21	4/21	1 (0.2–4.7)	1.00
Hypotension	7/21	8/21	1.3 (0.3–5.0)	1.00
Shock	0/21	0/21	1 (0.02–52.7	1.00
Somnolence	2/21	2/21	1 (0.1–7.8)	1.00
Dizziness	6/21	6/21	1 (0.3–3.8)	1.00
Syncope	1/21	1/21	1 (0.1–17.1)	1.00
Cranial nerve palsies	2/21	2/21	1 (0.1–7.8)	1.00
Acute respiratory failure	1/21	1/21	1 (0.1–17.1)	1.00
Acute myocardial injury	0/21	0/21	1 (0.02–52.7)	1.00
Acute renal failure	0/21	0/21	1 (0.02–52.7)	1.00
Rhabdomyolysis	3/21	3/21	1 (0.2–5.6)	1.00
Myoglobin (µg/L)	57 (26–143)	71 (40–148)		0.01
Thrombocytopenia (<150 × 10^9^/L)	17/21	17/21	1 (0.2–4.6)	1.00
Platelets (×10^9^)	70 (17–129)	70 (17–129)		0.07
D-dimer (>500 µg/L)	13/21	18/21	3.7 (0.8–16.6)	0.13
D-dimer (µg/L)	1476 (421–2673)	2001 (1773–9136)		0.01
INR (>1.3)	3/21	5/21	1.9 (0.4–9.1)	0.50
INR	1.15 (1.05–1.16)	1.16 (1.14–1.35)		0.01
Fibrinogen (<1.8 g/L)	0/21	1/21	3.1 (0.1–81.7)	1.00
Fibrinogen (g/L)	2.67 (2.40–3.25)	2.45 (2.20–3.15)		0.01
Disseminated intravascular coagulation	0/21	0/21	1 (0.02–52.7)	1.00
Procalcitonin (>0.24 µg/L)	2/21	8/21	5.8 (1.1–32.1)	0.13
Prokalcitonin (µg/L)	0.03 (0.02–0.08)	0.39 (0.03–1.52)		0.04
Leukocytosis (>11 × 10^9^/L)	12/21	17/21	3.2 (0.8–12.8)	0.06
Leucocytes (10^9^/L)	12.5 (8.4–15.2)	14.7 (12.1–18.4)		0.01

Legend: Atxs—ammodytoxins; INR—International normalized ratio; CI—confidence interval; OR—odds ratio.

**Table 3:** Therapy of *Vaa* envenomed patients.

Viperfav	
Time from bite to the first dose (median, IQR) (h)	4.0 (3.4–5.6)
Time from admission at the ED to the first dose (median, IQR) (h)	1.3 (0.4–2.3)
Adverse reaction to Viperfav	
Bradycardia	1/21
Hypotension	1/21
Anaphylactic reaction	0/21
Serum sickness	0/21
Other therapy	
Corticosteroids	2/21
Antihistamines	2/21
Analgesics	12/21
Antiemetics	12/21
Antibiotics	0/21
Low molecular weight heparin	0/21
Platelet transfusion	0/21
Red blood cell transfusion	0/21
Oxygen	0/21
Mechanical ventilation	0/21
Noradrenaline infusion	0/21
Outcome	
Length of hospital stay (median, IQR) (day)	2.5 (2–3.0)
Death	0/21

Legend: IQR—interquartile range.

**Table 4:** Pharmacokinetic values of *Vaa* venom-specific equine F(ab′)_2_ fragments (Viperfav) after intravenous administration in the patients envenomed by *Vaa*.

Pharmacokinetic parameters	Median (IQR)
*t*_max_ (h)	2.0 (1.2–3.7)
*c*_max_ (μg/mL)	196.4 (135.6–268.2)
*t*_1/2_ (h)	49.5 (26.1–68.0)
AUC_∞_ ((μg h)/mL)	9463.9 (6508.3–15,835.7)
AUC_0–*t*_ ((μg/mL)∙h)	5002.4 (4302.6–6854.4)
*V*_z_ (mL/kg)	36.1 (24.3–41.9)
MRT (h)	71.5 (38.5–105.3)
CL ((mL/h)/kg)	0.5 (0.4–0.7)

Legend: AUC_∞_, area under the serum concentration-time curve from time zero to infinity; AUC_0–*t*_, area under the serum concentration curve from time zero to *t*; CL, apparent total body clearance of the drug from serum; *c*_max_, maximum (peak) serum drug concentration; *t*_1/2_, elimination half-life; MRT, mean residence time; *t*_max_, time to reach maximum (peak) serum concentration following drug administration; *V*_z_, apparent volume of distribution during terminal phase. Data are presented as median and IQR.

## Data Availability

The original contributions presented in this study are included in the article. Further inquiries can be directed to the corresponding author.

## Abbreviations

The following abbreviations are used in this manuscript:

Atx	ammodytoxin
AUC_∞_	area under the serum concentration-time curve from time zero to infinity
AUC_0–*t*_	area under the serum concentration curve from time zero to t
ED	Emergency Department
CL	apparent total body clearance of the drug from serum
CI	confidence interval
*c*_max_	maximum (peak) serum drug concentration
INR	international normalized ratio
IQR	interquartile range
MRT	mean residence time
OR	Odds ratios
*t*_1/2_	elimination half-life
*t*_max_	time to reach maximum (peak) serum concentration following drug administration
*Vaa*	*Vipera ammodytes ammodytes*
*V*_z_	apparent volume of distribution during terminal phase
